# Kernel regression for fMRI pattern prediction

**DOI:** 10.1016/j.neuroimage.2010.03.058

**Published:** 2011-05-15

**Authors:** Carlton Chu, Yizhao Ni, Geoffrey Tan, Craig J. Saunders, John Ashburner

**Affiliations:** a Wellcome Trust Centre for Neuroimaging, UCL Institute of Neurology, London, UK; b ISIS group, Electronics and Computer Science, University of Southampton, Southampton, UK; c Section on Functional Imaging Methods, Laboratory of Brain and Cognition, National Institute of Mental Health, NIH, USA

**Keywords:** Kernel methods, Machine learning, Kernel ridge regression (KRR), fMRI prediction, Automatic relevance determination (ARD), Relevance vector machines (RVM), Regression, Multivariate

## Abstract

This paper introduces two kernel-based regression schemes to decode or predict brain states from functional brain scans as part of the *Pittsburgh Brain Activity Interpretation Competition* (PBAIC) 2007, in which our team was awarded first place. Our procedure involved image realignment, spatial smoothing, detrending of low-frequency drifts, and application of multivariate linear and non-linear kernel regression methods: namely kernel ridge regression (KRR) and relevance vector regression (RVR). RVR is based on a Bayesian framework, which automatically determines a sparse solution through maximization of marginal likelihood. KRR is the dual-form formulation of ridge regression, which solves regression problems with high dimensional data in a computationally efficient way. Feature selection based on prior knowledge about human brain function was also used. Post-processing by constrained deconvolution and re-convolution was used to furnish the prediction. This paper also contains a detailed description of how prior knowledge was used to fine tune predictions of specific “feature ratings,” which we believe is one of the key factors in our prediction accuracy. The impact of pre-processing was also evaluated, demonstrating that different pre-processing may lead to significantly different accuracies. Although the original work was aimed at the PBAIC, many techniques described in this paper can be generally applied to any fMRI decoding works to increase the prediction accuracy.

## Introduction

The purpose of this paper is to describe a general kernel regression approach to predict sensory and cognitive states from imaging data. Conventionally, functional imaging studies focus mainly on finding regions showing variation under controlled experimental stimuli. The most well-known technique is *Statistical Parametric Mapping* (SPM)(Friston et al., 1995). Under the assumptions of a general linear model (GLM), the time series at each voxel are modeled by a linear combination of experimental conditions and confounds (e.g. low frequency drifts). The statistical tests are later applied to the weighting of each experimental regressor to infer where contrasts (i.e. linear mixtures) of experimental effects can predict brain activity at each voxel. In other words, the objective is to detect regions of activation in the brain during tasks. Three-dimensional statistical maps are generated, which can be explained by contrasts of experimental conditions. The location of activation patterns provides insight into brain function. Alternatively, researchers may utilize the known function of those brain regions to make inferences about the particular experimental conditions.

In recent years, pattern recognition and machine learning methods have been used to predict or decode an experimental variable from high-dimensional imaging data. Not all methods are truly multivariate, as some still assume independence among voxels (Shinkareva et al., 2008). In general, these studies have well-controlled experimental stimuli, and the number of conditions are limited. The performance of the classification is determined by cross-validation, which involves partitioning the data into training and testing sets. The decoding machine, or classifier, is trained using the functional images and labels indicating the corresponding experimental condition. In the test phase, the classifier returns the predicted experimental conditions using test images as input. Because the true experimental conditions are known, the predictive accuracy can be calculated. Most of the published studies that applied pattern recognition to neuroimaging data involved block stimuli with categorical conditions, such as observing different categories of image stimuli or performing different tasks (Haynes and Rees, 2006, Haynes et al., 2007, Mourao-Miranda et al., 2006, Mourao-Miranda et al., 2007).

For classification problems, a distinction can be made between generative and discriminative models. A generative model would describe the entire probability distribution of each of the classes of data. An alternative is to use a discriminative model, which only needs to model the conditional probability of the class memberships (Ulusoy and Bishop, 2005). Generative models are usually not the most accurate approach to use for predicting. They require more hidden variables, so marginalization over higher dimensional probability densities is needed. Empirical evidence shows that discriminative pattern recognition models usually outperform generative models in terms of their predictive accuracy (Bishop, 2006) (although one could argue there is a fine distinction between a generative model of discriminative features and a discriminative model).

Discriminative models also allow different forms of question to be posed. For example, it becomes possible to estimate whether task C activates a network that is more similar to that activated by task A, or that activated by task B. By accurately characterizing the pattern of difference between A and B, it becomes possible to formulate questions in terms of this difference. More accurate characterizations of differences may also lead to tests with greater sensitivity. This has been demonstrated in studies that applied pattern recognition approaches to particular brain regions (Eger et al., 2008, Haynes and Rees, 2005). Such work has allowed differences to be detected that could not be found by mass-univariate approaches.

Most neuroimagers treat estimates of model parameters as the important findings, because these parameterize their model or question. Such studies generally involve simplified models, as these allow findings to be more easily visualised and explained.^1^ It is acknowledged that the models may depend on unlikely assumptions, but the benefits of adopting them should be evident from the literature. For example, mass-univariate statistical testing in SPM has proven to be a very powerful tool for visualising regional differences, despite the fact that it usually ignores the possibility of connections among different brain regions.

Hypothesis testing usually involves a comparison between two models (null and alternate), where the aim is to reject the null hypothesis if the alternative hypothesis models the data better. More recently, model comparison approaches have been introduced into the neuroimaging field (Friston et al., 2008), whereby a number of models are compared to identify those that best model the probability density of the data. In other words, the aim is to search for the most accurate model, where the measure of accuracy essentially concerns how well it would predict new data. The *Pittsburgh Brain Activity Interpretation Competition* (PBAIC) 2007 (http://www.lrdc.pitt.edu/ebc/2007/competition.html) allowed a comparison among a diverse range models for encoding the patterns of bold signal elicited in fMRI data by various tasks. As in the model comparison problem, it allowed the most accurate approach to be selected from a range of candidates. The benefits of PBAIC rest on being able to compare and contrast various models, of the same data, from different groups. In what follows we describe the models that we employed. The uniqueness of PBAIC 2007 was that it was formulated as a regression problem, rather than as one of classification. This is in contrast to most previous studies.

## Data acquisition and experimental design

This section will provide a summary of version 7 of the competition guidebook. For full details please refer to the guidebook, which can be downloaded from http://www.lrdc.pitt.edu/ebc/2007/materials.html. Briefly speaking, three subjects played a virtual reality game in a 3 T scanner (Siemens Allegra at Pittsburgh University). Each run was 20 min, and contained 704 scans with TR = 1.75 s. The game was a typical 3D first-person shooter (FPS) game, in which the subject navigated from the first person perspective. During the game, subjects were asked to fulfil certain tasks such as collecting weapons or fruits or taking pictures. There were intermittent periods of rests, during which the screen would turn gray with a white fixation cross, and the control from the joystick would also be paused. The task in the game also changed every few minutes from a pseudo instructor calling with a cell phone. When the subject was receiving the instruction, the control would be paused, but the subject could still see the scene. Objective feature ratings such as viewing faces, viewing dogs, and speed of motion were recorded automatically during scanning by joystick and eye trackers. However, subjective ratings were later obtained from subjects while viewing the record of their game play. To avoid confusion, “feature rating” here is analogous to the experimental conditions in standard fMRI experiments. It is also referred to as “rating” throughout this paper and in the competition guidebook. All ratings were rescaled in the 0–1 range. For the readers’ convenience, we present descriptions of the 13 mandatory feature ratings from the competition guidebook in Table 1, with our interpretations added. Sample pictures and movies of game play can also be found in the competition webpage. It is also possible to obtain the full video of game plays for all three players by contacting ebc@pitt.edu.

The scans of all three sessions were released, along with the objective and subjective ratings of only the first and second sessions (VR1, VR2). The aim of the competition was for entrants to predict the ratings from the third session (VR3). In order to score each team, the prediction accuracy of each rating was calculated from the Pearson's correlation between the predicted rating and the original rating convolved by a “canonical hemodynamic function.” In other words, the goal of the competition was to predict the convolved ratings, rather than the original ratings. There were a total of 13 mandatory ratings and 10 optional ratings for each team to predict. The final score of each team was calculated by averaging the *Z*-scores transformed from the correlation scores from each rating and each subject using Fisher's transform (Fisher, 1915). The motivation for using *Z*-scores came from the fact that correlations are very non-linear in terms of evidence against a null hypothesis of no association. Consequently, Fisher's transform makes un-remarkable correlations squash together, and 'remarkable' correlations extreme. This metric would also encourage teams to further improve their methods for those ratings that may have had the potential to achieve high predictive accuracies.

## Methods

A general processing pipeline is described with three major parts: (i) pre-processing, (ii) machine learning, and (iii) post-processing. However, not all the predictions of feature ratings followed this procedure; the process had some modifications to improve accuracy using knowledge about the nature of the ratings. These extra operations are explained at the end of the section.

### Pre-processing

The data were pre-processed using SPM5 (Wellcome Trust Centre for Neuroimaging, London, UK). All the scans were first realigned and resampled to remove the effects of subject motion. From a pattern recognition perspective, the variability arising from rigid body motion lies on a six-dimensional manifold embedded within the original 64 × 64 × 34 = 139264 dimensional space. Removing motion effects can be seen as a form of dimensionality reduction, which increases the similarity between scans.

**Fig. 1:**
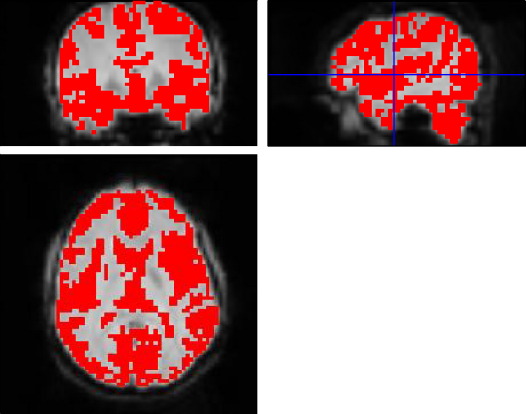
Gray matter mask overlaid on the original fMRI scan, where the segmentation was achieved by SPM5. Although part of the CSF was not cleanly removed, the masking did eliminate around 80% of the voxels from the original image.

To further reduce dimensionality, those voxels that were, *a priori*, considered non-informative were removed. Blood oxygenation level dependent (BOLD) signal change is generally believed to occur mainly in gray matter, as its major cause should be the local neuronal activity (Logothetis et al., 2001). Masks defining gray matter were generated for each subject by segmenting one of the fMRI scans (Ashburner and Friston, 2005) using methods implemented in SPM5. Voxels that did not contain gray matter were set to zero in all volumes of the time series (see Fig. 1). One practical reason for masking out non-gray matter tissue was to accelerate the speed of kernel generation. By masking out other tissues, only 20% of the whole image is used. It may also have been possible to co-register the anatomical image with the fMRI, and identify gray matter from this. Nevertheless, functional images tend to suffer from spatial distortions, especially in the frontal region due to the air in the frontal sinus, so it may not have been possible to accurately overlay gray matter masks derived from the anatomical scans.

For fMRI, signal changes due to brain activity tend to be slightly lower frequency over space than much of the noise. From a Wiener filtering perspective, the signal to noise ratio can be increase by spatially smoothing the scans. Empirically, we found that accuracy could be increased by convolving the scans with a 6 mm full width at half maximum (FWHM) Guassian kernel. Another reason for applying spatial smoothing was to suppress interpolation error due to image realignment of fMRI time series (Grootoonk et al., 2000).

Low frequency drift has often been reported in fMRI time series. This drift has been attributed to physiological noise or subject motion, but few studies have been done to test this assumption (Smith et al., 1999). The drift models currently dominating fMRI analysis are linear subspaces spanned by a set of polynomial or discrete cosine transform (DCT) basis functions (Friman et al., 2004, Tanabe et al., 2002). In our preliminary experiment, we observed that there was still a large amount of low frequency (0.0–0.0015 Hz) drift in the linearly detrended dataset provided by the PBAIC committee. Hence, we utilized DCT bases to eliminate additional low frequency drifts, as this is the default technique employed by the SPM software. Mathematically, for each voxel, the time series **v** = {*v*_*n*_}_*n* = 0_^*N*^^−^^1^ is collected from *N* time points and transformed into a frequency sequence **f** = {*f*_*l*_}_*l* = 0_^*N*^^−^^1^

(1) $$f_{l}=\sqrt{\frac{2}{N}}\sum_{n=0}^{N-1}v_{n}\cos[\frac{\pi }{N}(n+\frac{1}{2})l]\begin{array}{cc} & l=0,...,N-1 \end{array}$$

After pruning the low frequency drift (i.e. frequency components less than and equal to a particular number of minimum basis sets, say *L*), the detrended sequence **v̅** = {*v*_*n*_}_*n*= 0_^*N*^^−^^1^ is obtained by the inverse transform

(2) $$\bar{v_{n}}=\sqrt{\frac{2}{N}}\sum_{l=L+1}^{N-1}f_{l}\cos[\frac{\pi }{N}l(n+\frac{1}{2})]\begin{array}{cc} & n=0,...,N-1 \end{array}$$

Note that the DCT can be represented as a matrix multiplication. Let **G** be the *N x L* matrix with $g_{n,1}=\sqrt{\frac{2}{N}}\cos[\frac{\pi }{N}(n+\frac{1}{2})l]$, where *L* denotes the number of the minimum DCT basis which are meant to be removed. It can be shown that the detrending operation is

(3) $$\bar{v}=v-G(G^{T}v)=(I-GG^{T})v=Rv$$

where the matrix **R**=(**I**−**GG**^T^) is commonly known as the residual forming matrix (Friston et al., 2008). The operation can be applied to each voxel to generate the detrended scans. However, it is also feasible to use the more computationally efficient approach of directly detrending the linear kernel generated from the fMRI scans. Further details about the kernel will be explained in a later section, but generally speaking, a kernel is a matrix of similarity measures between each pair of scans. Suppose we define the input features **X** as an *N* x *D* matrix, which contains *N* input scans **X** = [**x**_1_,… **x**_*N*_] ^**T**^ and each vector **x**_*i*_ contains *D* voxels. Then, a linear kernel is defined as *K*(**x**_*i*_,**x**_*j*_) = **x**_*i*_^**T**^**x**_*j*_**,** and the *N* x *N* kernel matrix **K** = **XX**^T^ can be calculated.

(4) $$K_{\text{detrended}}=<X^{T}R,X^{T}R>=R^{T}XX^{T}R=R^{T}KR$$

**Fig. 2:**
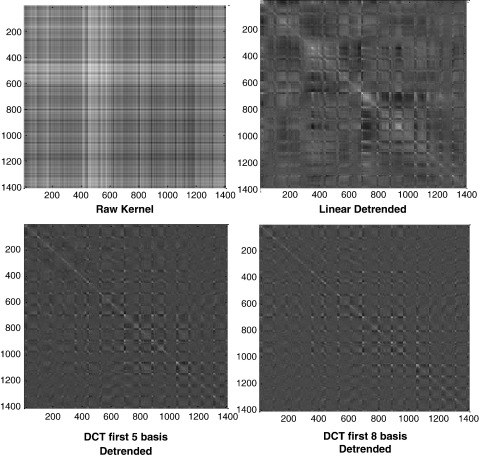
Linear kernel with no detrending and different degrees of detrending. The raw kernel without any temporal detrending and the linear detrended kernel seem to have less uniform intensities than those kernels with more low frequency components removed.

Notice the enormous reduction of computation as in general *D*>>*N*. It is also possible to apply other forms of detrending such as polynomial or piece-wise linear in this manner, as long as the detrending can be modeled as a matrix operation. So in general the residual forming matrix has the form. **R** = (**I**−**CC**^+^)**,** where **C** is any matrix of basis functions that model the drift. For example a quadratic basis set will be $C=\left(\begin{array}{ccc} 1 & 1^{2} & 1\\ \vdots & \vdots & \vdots \\ N & N^{2} & 1 \end{array}\right)$**,** and **C**^+^=(**C**^T^**C**)^−^^1^**C**^T^ is the pseudo-inverse of **C.** In our experiments, we used cross-validation to determine the optimal number of basis functions. Eight basis functions seemed to give robust results and is equivalent to a high pass filter with a cut-off of around 1/352 Hz. Detrending removes a large amount of variance from the fMRI kernel (see Fig. 2).

### Machine learning

Mathematically, we denote fMRI scans as {**x**_*i*_}_*i*= 1_^*N*^ which are embedded in a voxel feature space **x** ∈ ℜ^*D*^**.** *N* is the total number of time points, and the index *i* is ordered along the scanning sequence. The targets are the continuous variables of each feature rating, {**t**_*i*_}_*i*= 1_^*N*^**.** In the competition, there were 13 required ratings to predict, and 10 optional ratings. In our approach, each rating was treated independently.

For kernel regression methods, instead of evaluating the parameters in the space of input features, the problem is transformed into a dual representation. In this representation, solutions are sought in the kernel space, and the complexity is bounded by the number of training samples. This greatly reduces the computational complexity for high dimensional data (*D*>>*N*). In addition, with an appropriate kernel function, one can map the input space into a higher dimensional feature space (Shawe-Taylor and Cristianini, 2004). It is possible that the non-linear pattern in the original input space appears linear in this higher dimensional feature space. This is known as the kernel trick, and makes the linear regression or classification solution in the feature space equivalent to a non-linear solution in the original input space. Practically, some non-linear kernels can be directly computed from the original linear kernel. A commonly used example is the radial basis function (RBF)

$$\begin{array}{l} K_{\text{RBF}}(x_{i},x_{j})=\exp(-\gamma ||x_{i}-x_{j}|{|}^{2})\\ =\exp\{-\gamma (K(x_{i},x_{i})-2K(x_{i},x_{j})+K(x_{j},x_{j})\} \end{array}$$

Another example is the polynomial kernel

$$K_{\text{poly}}(x_{i},x_{j})={\left(\theta +x_{i}{}^{T}x_{j}\right)}^{d}={\left(\theta +K(x_{i},x_{j})\right)}^{d}$$

where *θ*, *d*, *γ* are functional parameters, which are often learnt through cross-validation. Alternatively, it is also possible to set the parameters by maximising their marginal likelihood (Rasmussen, 2006). In practice, we found linear kernel to be most robust when predicting most ratings except “Arousal” and “Valence.”

In the competition, we used two kernel regression methods: kernel ridge regression (KRR) and relevance vector regression (RVR).

#### Kernel ridge regression

Kernel ridge regression is the dual representation of ridge regression, which is sometimes known as the linear least square regression with Tikhonov regularization. The parameters from the input space are determined by minimizing a regularized sum of squares error functions given by

(5) $$\bar{w}=\underset{w}{\arg\min}\frac{1}{2}\sum_{i=1}^{N}{\left(w^{T}x_{i}-t_{i}\right)}^{2}+\frac{\lambda }{2}w^{T}w$$

where *λ* ≥ 0 is the regularization parameter, which is normally determined by cross-validation. Let **X** = [**x**_1_,… **x**_*N*_] ^**T**^, and **t**=[*t*_1_,…*t*_*N*_]^T^**.** If we take the derivative of the objective function with respect to the parameters **w**, we obtain the equations (Bishop, 2006, Shawe-Taylor and Cristianini, 2004)

(6) $$X^{T}Xw+\lambda w=(X^{T}X+\lambda I)w=X^{Τ}t$$

where **I** is the *D x D* identity matrix. In this case we can obtain the solution

(7) $$w={\left(X^{T}X+\lambda I\right)}^{-1}X^{Τ}t$$

In our problem, because the input dimension is in the order of tens of thousands, directly computing the matrix inversion becomes very difficult. Alternatively, we can rewrite Eq. (6) in terms of **w** to obtain **w** = *λ*^−^^1^**X**^T^(**t** − **Xw**) = **X**^T^**β**. This shows that **w** can be written as a linear combination of the training samples, **w** = ∑_*i* = 1_^*N*^*β*_*i*_**x**_**i**_, with **β** = *λ*^−^^1^(**t** − **Xw**). By substituting **w** with this new dual representation, it can be shown

(8) $$\begin{array}{l} \lambda \beta =(t-XX^{T}\beta )\\ \Rightarrow (XX^{T}+\lambda I)\beta =t\\ \Rightarrow \beta ={\left(K+\lambda I\right)}^{-1}t \end{array}$$

where **K=XX**^T^ is the kernel matrix as mentioned in the previous section. This formulation makes the computation much easier, as *K* is only *N* × *N*. To predict the output rating of a particular fMRI scan, the similarity measures between this scan and all the training scans are required. The prediction can be obtained by

(9) $$t_{*}=w^{T}x_{*}=\sum_{i=1}^{N}\beta_{i}K(x_{i},x_{*})$$

It is also possible to see ridge regression from a probabilistic perspective (Bishop, 2006, Hsiang, 1975). Applying Bayes’ rule leads to the posterior distribution of the parameters: *p*(**w**|**t**) ∝ *p*(**t**|**w**)*p*(**w**). If **w** has a shrinkage prior with zero mean and variance *α*^−^^1^, then the *maximum a posteriori* (MAP) solution is given by setting the derivative with respect to **w** to zero, to obtain

(10) $$w_{\text{MAP}}=\sigma^{-2}{\left(\sigma^{-2}X^{T}X+\alpha I\right)}^{-1}X^{T}t$$

where *σ*^2^ is the noise variance, assuming the noise is zero mean Gaussian. The MAP solution is equivalent to ridge regression where *λ* = *ασ*^2^. This interpretation, with some modification, leads to the following method.

#### Relevance vector regression

Relevance vector regression (RVR) (Tipping, 2001) is formulated in a Bayesian framework, while the general expression takes the form of a dual formulation and treats the kernel as a set of linear basis functions.

(11) $$t_{j}=\sum_{i=1}^{N}\beta_{i}K(x_{i},x_{j})+\beta_{0}=\sum_{i=0}^{N}\beta_{i}\phi_{i,j}$$

where *ϕ*_*i*,*j*_ is the element in the *N* × *N* + 1 ‘design’ matrix **Φ** = [1, **K**] with **K** denoting the kernel matrix and 1 denoting a column of ones.

Similar to the Bayesian view of ridge regression, each of the weights, **β**, are assigned a unique zero mean Gaussian prior. This differs from ridge regression, where all the elements of the weight have the same variance, *α*^−^^1^**.** The RVR models the prior of **β** with independent variance $p(\beta |\alpha )=\prod_{i=0}^{N}N(\beta_{i}|0,\alpha_{i}^{-1})$ and the solution involves optimizing the marginal likelihood (type-II maximum likelihood) with respect to the vector of hyper-parameters **α** and a noise variance *σ*^2^

(12) $$\begin{array}{l} p(t|\alpha ,\sigma^{2})=\int p(t|\beta ,\sigma^{2})p(\beta |\alpha )d\beta \\ ={\left(2\pi \right)}^{-\frac{N}{2}}|\sigma^{2}I+\Phi A^{-1}\Phi^{T}{|}^{-\frac{1}{2}}\exp\{-\frac{1}{2}t^{T}{\left(\sigma^{2}I+\Phi A^{-1}\Phi^{T}\right)}^{-1}t\} \end{array}$$

where **A** = diag(*α*_0_,...,*α*_*N*_) is a diagonal matrix. The objective of the optimization is to find the hyper-parameters, **A**, *σ*^2^, which maximize the “evidence” of the data. This can be achieved by expectation maximization. We refer readers to Bishop (2006) and Tipping (2001) for further details of this iterative procedure. In the training, some of the *α* will grow very large because *α* is the inverse of the variance of the prior of the parameter. A large value of *α* implies a small variance, a priori. Because the prior is zero mean, a parameter having extremely small variance results in its posterior probability being sharply peaked at zero. This property will prune out irrelevant columns of the design matrix, and is known as automatic relevance determination (ARD) (MacKay, 1995). Because the algorithm results a sparse solution, it means that only some of the training scans are used for prediction. Those scans are called “relevance vectors” which are analogous to “support vectors” in the SVM framework.

Predictions through RVR are given by

(13) $$t_{*}=\sum_{i=0}^{N}\mu_{i}\phi_{i,*}$$

where **μ** = *σ*^−^^2^(*σ*^−^^2^**Φ**^**T**^**Φ** + **A**)^−^^1^**Φ**^**T**^**t** is the posterior mean of the parameter **β**. Formulation (13) is nearly the same as Eq. (9), except in the RVR setup, a bias term was included.

### Post-processing

In most cases, the range of the raw feature ratings **z**_raw_, prior to convolution with the hemodynamic response function (HRF), was between zero and one. To utilize this known information, a constrained de-convolution strategy was applied (Gitelman et al., 2003). The “canonical HRF,” which the competition used to convolve the raw ratings with, was generated. The convolution can be implemented as a matrix multiplication of the raw rating by a toeplitz matrix **H**,**t** = **Hz**_raw_. The objective is to recover the raw rating **z**_raw_ fulfilling the constraints by minimizing the sum of square loss between the re-convolved solution **Hz**_raw_ and the predicted rating t_*_. Quadratic programming (the same optimization used by support vector machines) was used to de-convolve the HRF from the predictions (t_*_) by

(14) $$\underset{z_{\text{raw}}}{\arg\min}\{{(Hz_{\text{raw}}-t_{*})}^{T}(Hz_{\text{raw}}-t_{*})\}=\underset{z_{\text{raw}}}{\arg\min}\{z_{\text{raw}}^{{}^{T}}H^{T}Hz_{\text{raw}}-2t^{T}Hz_{\text{raw}}\}$$

subject to the constraints 0 ≤ **z**_raw_ ≤ 1**.**

**Fig. 3:**
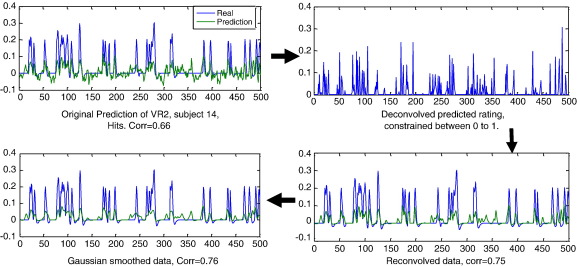
The graph shows the improvement of prediction accuracy achieved by constrained deconvolution, followed by re-convolving and smoothing. On the top left graph is the blue line showing the true rating, and the green line shows the prediction. The HRF was deconvolved from this prediction, under the constraint that the results fall between zero and one (top right graph). On the bottom right, the deconvolved prediction is re-convolved with the canonical HRF. The correlation had substantial improvement. A final smoothing step (bottom left) further increased the correlation.

The new predicted rating is then **t** = **Hz**_raw_. After the re-convolution, the rating was further smoothed by a Gaussian kernel with a FWHM of three time points. This FWHM was determined empirically (Fig. 3).

#### Feature selection with prior knowledge

*Ugly Duckling Theorem* (Duda et al., 2001, Watanabe, 1970) tells us that prior knowledge is essential for quantifying the similarity between things, so knowledge about human brain function was used to further increase the signal to noise ratio and suppress those features that were believed, *a priori*, to be less informative. It is known from the functional brain mapping literature that some cognitive functions and sensory perceptions are regionally localized. Hence, masks were used to weight the kernels when predicting the two feature ratings: “dog barking” and “interior or exterior of the building.” It was believed that most of the fMRI pattern resulting from the barking sound would be localised in auditory cortex. Similarly, the major discrimination between the inside and outside of the buildings would be the illumination differences. Therefore, a mask of visual cortex could mask out a large amount of irrelevant signal.

**Fig. 4:**
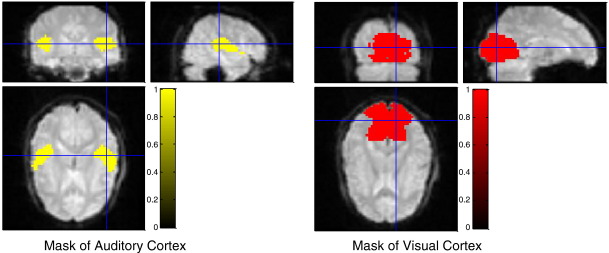
Regional masks generated from functional regions of the brain. The masks were downloaded from the McConnell Brain Imaging Centre. The auditory mask improved the prediction of “Dog,” and the visual mask improved the prediction of “Exterior/Interior” (inside or outside the buildings).

In order to generate the mask of functional regions for all three subjects, the cytoarchitectonic maps of visual and auditory in stereotaxic space were first downloaded from the McConnell Brain Imaging Center (http://www.bic.mni.mcgill.ca/cytoarchitectonics/). Then the deformation field generated by the normalization routine in SPM5 was used, but rather than warping the individual to the MNI space, the cytoarchitectonic maps in MNI space were warped so that they overlay the individual subject's fMRI data. Finally, a threshold of 0.3 was used to convert the probability maps into binary masks (Fig. 4).

### Post-processing for predicting “Instruction”

Because the competition scoring was based on *Z*-scores, we found that increasing a correlation from 0.8 to 0.9 resulted in three times as much improvement in the final scores as raising a correlation from 0.2 to 0.3. The goal was therefore to focus attention on those ratings that could be predicted reasonably well, and improve them further.

**Fig. 5:**
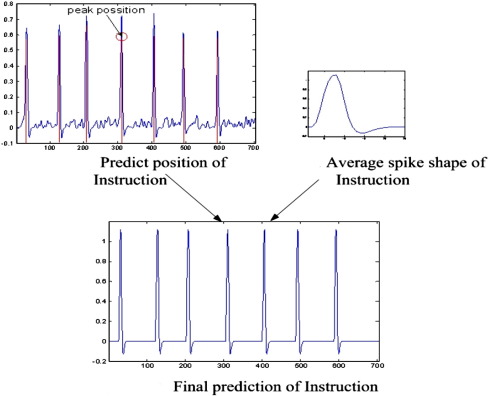
Model fitting approach to boost the prediction of “Instruction” to a correlation of 0.99. The top left graph shows the original prediction. The average shape of the response to “Instruction” was generated to fit the raw prediction.

It was observed that the “Instructions” ratings had seven spikes, which all had similar shapes across all subjects and sessions. It became apparent that an ad hoc model fitting strategy could be used to further improve what were already high correlations. Firstly, kernel regression was applied to predict the rating, and then the prediction was convolved with the model shape, which was generated by averaging all the spikes in all sessions of all subjects. This is equivalent to match filtering, and the peak values in the convolved ratings indicate the location where the average shape fits best. After finding the estimated peak location, the average shape was inserted (Fig. 5). Without this procedure, the correlation of the predicted rating was 0.8, whereas by adopting it, the final correlation reached 0.988, which increased the *Z*-score from 1.0986 to 2.555.

#### Temporal shifting

Unlike most conventional fMRI studies, which use controlled external stimuli, some of the ratings were self-paced. These included “hits” and “velocity,” which were believed to have different HRF delays from the canonical HRF. The stringent way should be to train with ratings convolved with differently specified HRFs, but there are at least five parameters to adjust for generating a HRF using double gamma functions. For reasons of generalization and robustness, we simply applied forward or backward shifts by discrete numbers of time points (scans). The predicted rating was later inversely shifted.

## Results and discussion

**Fig. 6:**
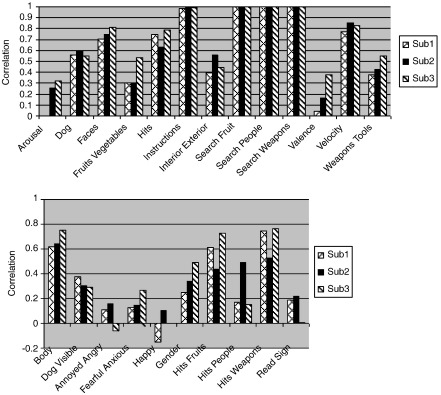
Prediction accuracy of our final (third) submission for all three subjects. The top graph shows the compulsory feature ratings, and the bottom graph shows the optional feature ratings.

Overall, no single method had the best performance across all ratings. Fig. 6 shows the correlations achieved by our final submission, which was the combination of best results from the first and second submission. For the first submission, kernel ridge regression was used, whereas for the second submission, most ratings were predicted with RVR. Individual differences also appeared to play a large part in how well we were able to predict ratings, as the a subject who performed worse or better in one rating also performed consistently worse or better in other ratings. For example, subject 1 had the worst prediction accuracy (average *Z*-score 0.980), especially for emotional and subjective ratings such as “Arousal,” “Valence,” “Fearful/Anxious” and “Happy.” Subject 3 had the best overall prediction accuracy, with an average *Z*-score of 1.142. The variation of prediction accuracy for each rating across all subjects is quite consistent, i.e. subject 1 is often the worst; this implies that accuracy is influenced by subject-specific issues. This may relate to concentration, but was most likely due to motion in the scanner. By inspecting the movement parameters generated from the realignment procedure, subject 1 clearly showed more translation and rotation than subjects 2 and 3. Our ability to predict particular ratings was clearly higher for objective ratings such as instructions, velocity and faces, than it was for subjective ratings. This may be related to the reliability of the reported ratings (many of the subjective ratings were made at a separate occasion based on episodic recall of how they felt), and that this will improve if real-time measures such as skin conductance and heart rate or subjective ratings between each block were used instead. Among objective ratings, we were able to best predict those that involved attention or required a response on the part of the subject. Thus “Instructions” required the subject to attend and comprehend, while “velocity” and “hits” required a motor response from the participant. These were followed by anthropomorphic objects such as faces and bodies. Indeed, the highest of the optional ratings (lower graph in Fig. 6) involved humans, such as looking at bodies and discrimination of gender. Hit ratings were highest for people. We believe this is because humans have particular object expertise in humans and recruit additional cross-modal systems such as the mirror system in the representation beyond the primary sensory modalities.

**Fig. 7:**
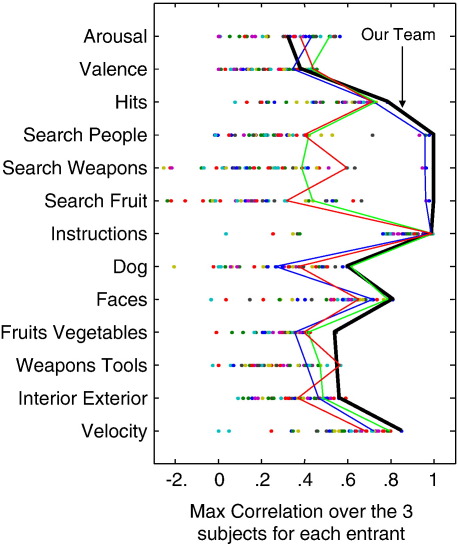
This is the maximum correlation over the three subjects for each team. The result of our team is shown in the thick line. Our team predicted well for most ratings, except “Arousal” and “Valence.” This figure is originally from http://www.ebc.pitt.edu/2007/Slides/All.ppt.

As the 1st place winner in 2007 PBAIC competition, our final competition score was 0.785 which was a substantially higher than other groups. Generally speaking, our team predicted all the objective ratings well within the top 5% of the maximum correlation for the entry, and we had the best prediction over the three subjects for “Hits,” “Search People,” “Search Weapons,” “Search Fruit,” “Faces,” “Fruits Vegetables,” and “Velocity” (Fig. 7) Some readers may be puzzled why our team achieved near perfect prediction for “Search People,” “Search Weapons,” and “Search Fruit.” It was actually based on an ad hoc procedure which exploited flaws in the competition design. Further details may be found in the Supplementary material. Although our methods predicted objective ratings well, it did not perform well for the subjective ratings, which were “Arousal” and “Valence.” It is probably because our team used the entire gray matter, and results from groups who did feature selection seemed to be more accurate for those two ratings. We used a linear kernel to predict most ratings, except “Arousal” and “Valence,” which were predicted by RBF kernels. Cross-validation showed that linear kernels performed relatively poorly for those two ratings compared with RBF kernels. Sometimes, linear kernels even yielded negative scores in cross-validation. We suspect it is because linear methods are only able to model a single mode of difference, whereas non-linear models can potentially model multiple modes of variability. This may indicate that these states may be represented in the brain by several alternative networks of activity, rather than a single consistent pattern of differential activity. In addition, the reason we favoured RBF kernels rather than polynomial kernels is that polynomial kernels require two parameters, which increase the complexity of the model. Based on Ockham's Razor (Duda et al., 2001), when both non-linear kernels achieve equivalent performance, we tended to select the one requiring fewer parameters.

According to the competition committee, more than 40 teams submitted their final predictions. A list of participants can be found at http://www.lrdc.pitt.edu/ebc/2007/2007.html. Some teams also submitted their methodological reports on the website. The most popular approaches were RVR, SVM, ridge regression, and neural networks. There were also some potentially very interesting techniques, such as elastic net regularization, fuzzy ARTMap, and functional data analysis.

In addition to winning the competition, our team made some original contributions to the methodological development of neuroimaging. We were the first group to apply RVR to fMRI data in PBAIC 2006. This sound approach also caught the attention of group from University of Maastricht, as they also applied RVR in PBAIC 2007, and won the 2nd position. The other major contribution would be the “kernel detrending” (Eq. (4)), which is computationally efficient. Although, similar formulations were also mentioned in (Friston et al., 2008), we realized it can also be applied to the kernel formulation. Kernel detrending is a general process for kernel methods, so people who apply SVM or other kernel algorithms can also utilize this approach.

### Relevance vector machine vs. kernel ridge regression

On average, kernel ridge regression (KRR) performed slightly better than relevance vector regression (RVR), but the results are mostly within 10% of each other. In Table 2, we compared KRR and RVR with five different feature ratings for subject 3, using a linear kernel. In addition, the sparseness of RVR (percentage of the training scans contributing to the prediction) is presented in Table 3. As we observed, KRR performed slightly better for most ratings. It is possible that sparse representations may not fully utilize all the information in the training set; hence pooling all the training scans would probably estimate the variance component more accurately. However, from Table 3, RVR required less than 25% of the training data to make predictions, with less than a 10% sacrifice of accuracy. For ratings that could be predicted well, such as “Velocity” and “Faces,” the differences between RVR and KRR are only about 1%. This sparsity may be due to consistent activation patterns in the brain during the same ratings; hence the regression machine only required a subset of training data to represent such patterns.

**Fig. 8:**
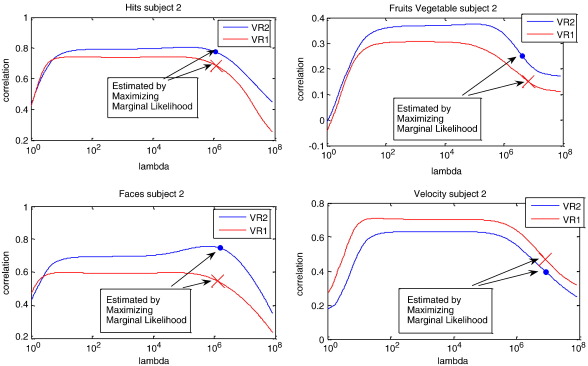
Cross-validation results of subject 2 using kernel ridge regression (KRR) to predict four ratings – “Hits,” “Fruits Vegetable,” “Faces,” and “Velocity.” The horizontal axis indicates different amounts of regularization for KRR. The plotted line of VR1 indicates the prediction of the first session by training from the second session, and vice versa. The dot is the prediction for VR1 estimated via maximizing of marginal likelihood, and the cross is the prediction for VR2.

Unlike RVR, where the hyper-parameters are determined through maximization of marginal likelihood, the regularization parameter for KRR was determined empirically by cross-validation. In Fig. 8, the correlations obtained by training with virtual reality (VR) game 1 then testing on VR game 2, and vice versa, were evaluated with different regularization parameter for four feature ratings. The graph shows that the correlation reaches a plateau with the regularization roughly between 10^2^ and 10^5^. Alternatively, it is possible to optimize the regularization parameter by maximizing the marginal likelihood, which is equivalent to the Gaussian Processes (GP) approach (Bishop, 2006, Rasmussen, 2006).

(15) $$\begin{array}{l} p(t|\theta )={\left(2\pi \right)}^{-\frac{N}{2}}|C{|}^{-\frac{1}{2}}\exp\{-\frac{1}{2}t^{T}C^{-1}t\}\\ C=\theta_{1}I+\theta_{2}K \end{array}$$

This equation is a generalized version of Eq. (13) for RVR, and the vector **θ** contains hyper-parameters, which the algorithm optimizes. The regularization parameter for ridge regression is simply obtained by *λ* = *θ*_1_/*θ*_2_. Intriguingly, in Fig. 8, it seemed the regularization determined by maximizing marginal likelihood was over-regularized, and the results were not very desirable. This demonstrates the importance of well specified models to the application of Bayesian techniques, and could explain the better performance of KRR with respect to RVR. If a good model structure is not accurately known, then cross-validation may allow more accurate tuning of various hyper-parameters than the Bayesian evidence framework. In our case, the less accurate solution found by GP may be due to several factors. First, no temporal autocorrelations were modeled, whereas the actual noise for fMRI data is not independent and identically distributed (iid). The evidence framework may have found more accurate hyper-parameter estimates if auto-correlations had included in the model. Second, the objective function for maximizing the marginal likelihood is based on sum of the squares differences, which may have different characteristic from Pearson's correlation coefficient. Third, a proper covariance matrix **C** should contain a constant term **C** = *θ*_1_**I** + *θ*_2_**K** + *θ*_3_. Empirical investigations showed that including the constant term improved the correlation to around the same accuracy as the plateau in the cross-validation plot.

### Importance of pre-processing

**Fig. 9:**
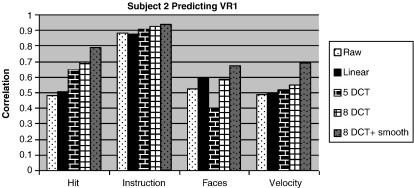
The graph shows the prediction accuracy for subject 2 for predicting the first session of the VR game by training with data from the second session. Different pre-processing settings were used.

We believe that one of the contributing factors for our team's success was the spatial and temporal pre-processing. Spatial smoothing and temporal detrending have been shown to change the results of SPM, as well as the prediction accuracy (LaConte et al., 2003, LaConte et al., 2005, Strother, 2006, Tanabe et al., 2002). Visual inspection of the kernels (Fig. 2) shows that they can appear to be rather patchy. The raw kernel, without any temporal detrending, and the linear detrended kernel, both have less uniform intensities than those kernels with more low frequency components removed. Some of the pattern in the kernels is due to the fixation periods. One major reason why temporal detrending is important is because scans from the all three games were combined together. In other words, all the scans were assumed to be collected in the same session with the same intra-sessional variance. If the low frequency components dominated the major variance components, i.e. the first few principle components, the signals due to brain activations would be reduced. In Fig. 9, the results of cross-validation performed with four ratings for subject 2 are shown. Three different degrees of detrending were compared, as well as the result of high detrending plus spatial smoothing. These comparisons clearly show that higher detrending improves the prediction accuracy – except for “Faces.” In general though, detrending with eight DCT bases, with spatial smoothing (6 mm FWHM Gaussian) gave the best results for all four ratings. The improvement was most prominent for “Hits” and “Velocity.” For “Hits,” the correlation improved from 0.5 with no detrending or smoothing at all, to 0.8. For “Velocity” the correlation rose from 0.5 to 0.7 after high detrending and spatial smoothing. Fig. 7 shows that our strategy performed better than other groups for those two ratings.

### Visualization of the fMRI map

**Fig. 10:**
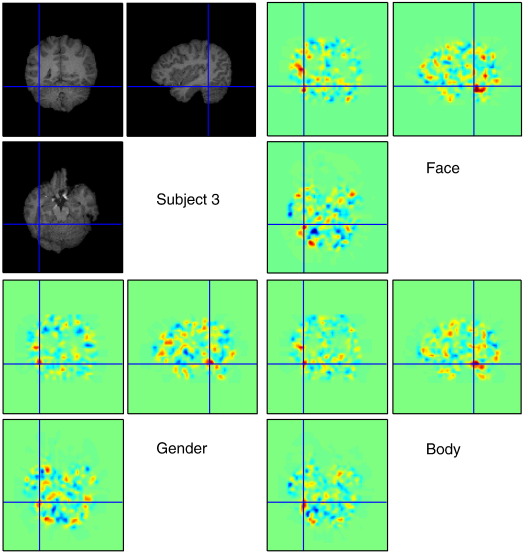
The weight map, or weight vector, in feature space shows positive weightings in posterior superior temporal sulcus (pSTS) for predicting “Gender,” “Faces,” and “Body” of subject 3. The red indicates positive weightings and the blue indicates negative weightings.

If a linear kernel is used, it is possible to create a single summary map for a particular rating. In our experiment, this map was created directly from the weights in the feature space. **w** = ∑ _*i*= 1_^*N*^*β*_*i*_**x**_**i**_ = **X**^**T**^**β**, which is a linear combination of all the training scans for KRR, or only the relevant scans (i.e. with non-zero weights) in the RVR framework. In other words, the prediction can be calculated from the dot-product of this weight map and the scan at a particular time point, *t*_*j*_ = < **w**,**x**_*j*_> = **w**^**T**^**x**_*j*_**.** If **β** was learnt from the detrended kernel, then the weight map is **w** = **X**^**T**^**Rβ**. Fig. 10 shows the weight maps for “Faces,” “Bodies” and “Gender,” which are surprisingly similar. Common areas include those known to be activated when viewing human/humanoid biological motion, particularly the posterior superior temporal sulcus (pSTS), extending to the ventral visual streams bilaterally corresponding to the fusiform and middle occipital gyri (Morris et al., 2005).

#### Effect of temporal shift

As mentioned in the Methods section, temporal shifting was employed to account for shorter hemodynamic delay. It was found, by cross-validation, that shifting the original training targets by one scan (1TR) earlier would yield more accurate predictions. Table 4 shows the results of cross-validation for “Hits,” “Velocity,” and “Faces.” In these three feature ratings, only “Hits” did not show consistent improvement across all three subjects. “Velocity” and “Faces” both showed increasing accuracy for all subjects. This led us to ask why measured brain activity preceding an event would appear to be more predictive. It is possible that the regions involved for those two ratings had an HRF that was considerably shorter than the HRFs of regions involved in discriminating other ratings. This seems unlikely, however, as the most relevant or highly weighted voxels were distributed across several regions of the brain and, as we subsequently show, there was a spatial shift in the most heavily weighted voxels accompanying the temporal shift in prediction. The alternative explanation is that brain activity preceding the event reflects what is subsequently recorded.

**Fig. 11:**
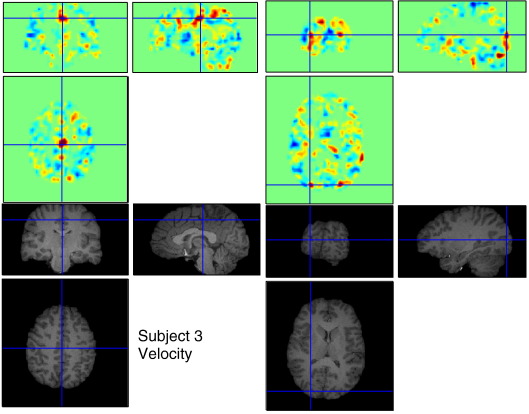
The weight map of “Velocity” for subject 3. There contain strongly positive weightings in both motor and visual areas.

The “Velocity” rating is related to the amplitude of joystick movement, so the involvement of processes underlying voluntary motor control would be expected. Motor preparation, or the readiness potential, has been known to precede onset of voluntary motor execution by over a second. This would conceivably correspond to the period of 1 TR. As expected, inspection of the weight vector in voxel space (Fig. 11) shows that the motor areas around M1, the supplementary motor area and cerebellum had activity positively weighted with ratings (Cunnington et al., 2002). In addition, dorsal visual areas over the occipital and parietal cortices may be associated with the visual effect of the moving background and motor attention (Rushworth et al., 2001).

PBAIC provided an objective measure of accuracy with which to compare approaches for modeling fMRI data, but with more challenging patterns of noise than is typical for most fMRI studies. Accurately predicting a brain state requires a model of the pattern of activity that differentiates the brain state from others. It is interesting to note that multivariate (rather than mass-univariate) methods were able to model these patterns of brain activity most accurately. The competition also demonstrated that utilizing prior information, such as removing low frequency drifts or selecting functional regions, is the key to achieving good empirical success. Compared to these pre-processing issues, the choice of multivariate learning algorithm appears to have relatively little effect. Other useful findings from the results of PBAIC2007 were that the predictability of different ratings can be used to determine the robustness of fMRI patterns for the corresponding cognitive processes. The fact that some processes were better encoded using non-linear models may suggest that what are often considered to be the same cognitive state, may in fact be encoded in the brain by a multitude of different patterns of activity. This knowledge may serve as guidance for brain computer interfaces or real-time fMRI. The scripts and learning algorithm we implemented for the competition are available from http://fim.nimh.nih.gov/people/chiayueh-carlton-chu.

## Figures and Tables

**Table 1:** Description of feature ratings.

Feature rating	Description	Rating type	Addition interpretation and findings from our team
Arousal	How much does what is going on in the scene affect how calm the subject is.	Subjective (Discrete, 5 levels)	
Valence	How positive or negative is the environment.	Subjective (Discrete, 5 levels)	
Hits	Times when subject correctly picked up fruit or weapon or took picture of a pierced person.	Computed from VR software (Binary, 0 or 1)	It involved different cognitive functions. The subject had to first control the joystick to aim the object (motor and visual), and then click the button to pick up object or take picture (motor). A high-pitched ring was accompanied with a successful hit (auditory).
Search People	Times when subject searched for pierced people.	Computed from VR software (Binary, 0 or 1)	During these “periods,” subjects took pictures of people, i.e. highly correlated with the optional rating “hits people.”
Search Weapons	Times when subject searched for weapons.	Computed from VR software (Binary, 0 or 1)	During these “periods,” subjects took weapons from the ground, i.e. highly correlated with the optional rating “hits weapons.”
Search Fruit	Times when subject searched for fruits.	Computed from VR software (Binary, 2 levels, 0 or 1)	During these “periods,” subjects took fruits from the ground, i.e. highly correlated with the optional rating “hits fruit.”
Instructions	Times when task instructions were presented.	Computed from VR software (Binary, 0 or 1)	They were strong auditory stimuli, as the volumes were relatively high.
Dog	Times when dog was audible to the subject.	Computed from VR software (Binary, 0 or 1)	They were weak auditory stimuli, as the volumes were relatively moderate.
Faces	The degree to which faces of a pierced or unpierced person were visible to the subject.	Computed from eye tracker (Continuous)	Empirical results show that using whole brain achieved better prediction accuracy than using face selective areas or visual cortex alone.
Fruits Vegetables	The degree to which fruits or vegetables were visible to the subject.	Computed from eye tracker (Continuous)	
Weapons Tools	The degree to which weapons or tools were visible to the subject.	Computed from eye tracker (Continuous)	
Interior Exterior	Times when subject was inside a building (1 subject was inside, 0 = subject was outdoors).	Computed from VR software (Binary, 0 or 1)	We found that visual cortex was involved in this stimuli, because the overall luminance was generally higher outdoors than indoors.
Velocity	Velocity of the subject moving in the VR world but not interacting with an object.	Computed from VR software (continuous)	This condition should involve motor and visual functions, because subjects controlled the joysticks to move, and moving would cause motion in vision.

**Table 2:** Comparing the accuracies of KRR and RVR for predicting the third session of subject 3.

	Velocity	Hits	Weapons Tools	Fruits Vegetable	Faces
Kernel ridge regression	0.8277	**0.7835**	**0.5470**	**0.5366**	**0.8091**
RVR	**0.8309**	0.7552	0.4998	0.4955	0.7995

**Table 3:** Sparsity measures for RVR (percentage of the training scans contributing to the prediction).

	Velocity	Hits	Weapons Tools	Fruits Vegetable	Faces
RVR	21.3%	24.4%	22.8%	23.3%	18%

**Table 4:** Cross-validation results for “Hits,” “Faces” and “Velocity,” obtained by shifting the training target one time point earlier.

	Subject 1		Subject 2		Subject 3	
Predict VR1	Predict VR2	Predict VR1	Predict VR2	Predict VR1	Predict VR2
*“Hits”*						
Original	0.5873	0.6861	**0.7427**	**0.8030**	0.6019	**0.7551**
Apply shift	**0.6094**	**0.7272**	0.735	0.8	**0.6096**	0.7341

*“Faces”*						
Original	0.5538	0.5436	**0.**589	**0.**7521	0.8313	**0.**8706
Apply shift	**0.5549**	**0.553**	**0.7114**	**0.8155**	**0. 8328**	**0.8859**

*“Velocity”*						
Original	0.7217	0.7207	0.7010	0.6347	0.664	0.7022
Apply shift	**0.7432**	**0.7312**	**0.7481**	**0.6464**	**0.7059**	**0.7508**

## Footnotes

1 This simplification is distinct from Ockham's Razor, and the bias-variance trade-off involved in model selection.
