# Supplementary material for: Kernel regression for fMRI pattern prediction
Source: Neuroimage. 2011 May 15;56(2-10):662–73. doi: 10.1016/j.neuroimage.2010.03.058 (PMC3084459; doi:10.1016/j.neuroimage.2010.03.058)
Supplement: Supplementary file 1 [file mmc1.doc]

# Supplementary Material

## Knowledge integration for predicting “Search Weapons, People, and Fruit”

According to the report of the competition in 2007, the maximum correlations achieved by “search fruit”, “search weapons”, and “search people” all exceeded 0.99. However, those three ratings were not predicted in the same way as other ratings, and the “search” ratings in the first and second session of the virtual reality games weren’t actually used for training the regression machine. To achieve such high accuracy, prior knowledge was utilised to arrive at a solution from prediction of more accurate ratings. From observation, a few repeating patterns were found in the design of the virtual reality (VR) game. These could be seen as design weaknesses, which we exploited as far as possible.

1. Each session of the game was cut into seven slots, each of which started at the “instruction” and ended with the “instruction” except the last slot.
2. For each request of searching (fruit, weapons, and people), it appeared after the instruction and occupied exactly four arbitrary slots at each session.
3. Each slot had at least one request.
4. The requests to search the three categories were the same for all subjects.
5. The optional ratings of “hit (people, weapons, and fruit)” only appeared when the slot had the same category of search request, otherwise the “hit something” rating would be zero for the whole slot. For example, if during a particular time slot, the search requests were “search people” and “search weapons”, then the rating of “hit fruit” would be zero for the entire slot.

Cross-validation showed that training with the “search (people, weapons, and fruit)” rating returned very low accuracy, with correlations of only about 0.2. However, predicting “hit (people, weapons, and fruit)” could achieve correlations of 0.4~0.5 for at least one of the subjects. Hence, we used the prediction of “hit something” to predict “search something”. For example, if during one slot, the “hit people” rating is non-zero at some point, there would definitely be a “search people” request in that same slot. Although the prediction of “hit something” would contain noise, the strength of the noise was believed to be lower than that of “true hits”. Therefore, by thresh-holding the prediction and only keeping high peaks, most noise may be pruned out, but enough true “hits something” may be kept to infer which slots had the particular category of search request. Motivated by this observation and previous findings, the procedure can be summarized by the following steps: (Figure 6)

1. Predict “hit (people, weapons, and fruit)” for three subjects.
2. Prune most of the points and only keep some high value peaks (top 20%).
3. Count how many peaks are in each slot. For each “search something” request, we found the four slots containing the highest peak counts. A majority vote was used for cases where the peak counts differed over the three subjects. The rating of the “search something” was then set to one during the four slots.
4. Finally, convolve the predicted block with the canonical HRF.


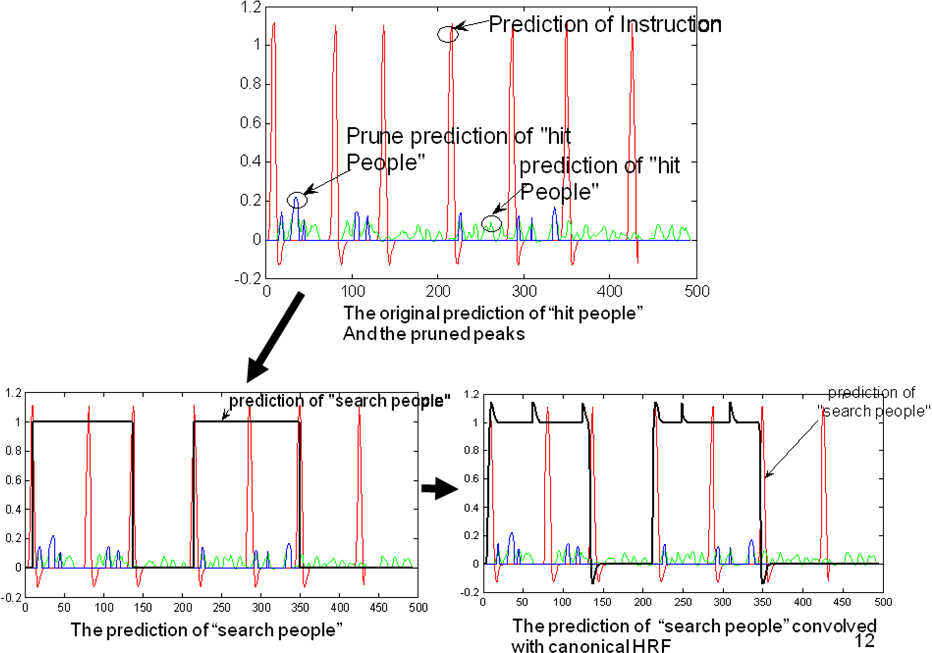


**Figure S1:** Prediction of “Search People” using prediction of “Hit People”. The red spikes are the prediction for “Instruction”. For simplification, the fixation (resting) period was removed. The “Instruction” divided each session into seven slots. The prediction of “Hit People” is shown in green, and the thresholded version of “Hit People” is shown in blue. Four out of the seven slots were selected, based on the strength and frequency of the thresholded “Hit People”. In this case they are slots 1 2, 4, and 5, then the rating was set to 1 for time points in those four slots. The prediction was furnished by convolved with the HRF.
